# Dr. Paris's Pharmacologia, or History of Medicinal Substances, with the View to Establish the Art of Prescribing Extemporaneous Formulæ, &c. &c.

**Published:** 1820-06-01

**Authors:** 


*Pharmacologia; or, the History of Medicinal Substances, with a View to establish the Art of prescribing and of composing extemporaneous Formulæ upon fixed and Scientific Principles: illustrated by Formulæ, in which the Intention of each Element is designated by Key Letters.* By JOHN AYRTON PARIS, M. D. F. L. S. Fellow of the Royal College of Physicians London, &c. &c. One vol. Octavo, pp. 432, London, 1820. Third Edition, very considerably enlarged.

"*Quis Pharmacopæo dabit leges, ignarus ipse agendorum?....Vix profecto dici potest, quantum hæc ignorantia rei medicæ inferat detrimentum.*" GAUB. METHOD. CONCINN. FORMUL.

As the finest army may be rendered ineffective by bad arms or ammunition, so the ablest physician may be baffled in his therapeutics by an unskilful combination of the remedies which he prescribes. We hardly know which is the more dangerous practitioner—the pathologist without accurate pharmaceutical knowledge, or the pharmacopolist without sound principles of pathology. This much is certain, that all branches of medical science must be *studied*, however we may settle the divisions of *practice* afterwards. We are highly gratified, then, when we see a well-educated physician direct, occasionally, a more than ordinary proportion of attention to some individual branch of our science, for it is not by one general consentaneous movement that the science can advance, as a whole, and much less by the power of one man acting equally on all its parts—*Non omnia possumus omnes.*

Three impressions of a *regular* medical work afford unequivocal testimony of public approbation; but this third edition of the Pharmacologia is so enlarged and improved, as to be, in fact, a new work. Although incapable of being regularly analysed, we conceive it our duty to lay such a delineation of the nature of the publication before our readers, as may enable them to form some estimate of its importance.

"The term Pharmacologia, (says Dr. Paris) as applied to the present work, may therefore be considered as contra distinctive to that of Pharmacopœia; for while the latter denotes the processes for preparing, the other comprehends the scientific methods of administering medicinal bodies, and explains the objects and theory of their operation." *Pref.*

Under the history of each article he has endeavoured to concentrate all that is required to be known for its efficacious administration, such as—

"1. *Its sensible qualities.* 2. *Its chemical composition*, or the constituents in which its medicinal activity resides. 3. *Its relative solubility in different menstrua, and the proportions in which it should be mixed, or combined with different bodies, in order to produce suspension, or saturation.* 4 *The incompatible substances*, that is to say, those substances which are capable of destroying its properties, or of rendering its flavour or aspect, unpleasant or disgusting. 5. *The most eligible forms in which it can be exhibited.* 6 *Its specific doses.* 7. *Its medicinal uses, and effects.* 8. *Its officinal preparations.* 9. *Its adulterations.* That such information is indispensible for the elegant and successful exhibition of a remedy, must be sufficiently apparent; the injurious changes and modifications which substances undergo, when they are improperly combined by the ignorant practitioner, are not, as some have supposed, imaginary, the mere *deliramenta doctrinæ*, or the whimsical suggestions of theoretical refinement, but they are really such as to render their powers unavailing, or to impart a dangerous violence to their operation. '*Unda dabit flammas et dabit ignis aquas.*" *Preface.*

The adulteration of medicines, now too shamefully practised, is properly taken notice of by our author, and the formulæ of the principal *quack medicines* have been appended in notes.

An historical introduction, comprehending the substance of two of the lectures read last year before the Royal College of Physicians, has been prefixed to the work, which is divided into two separate and very distinct parts, the *first* comprehending the principles of the art of combination—the *second*, the medicinal history, and chemical habitudes of the bodies which are the subjects of such combination.

"These comprise every legitimate source of instruction, and to the young and industrious student, they are at once the LOOM and the RAW MATERIAL. Let him therefore abandon those flimsy and ill adapted textures, that are kept ready fabricated for the service of ignorance and indolence, and by actuating the machinery himself, weave the materials with which he is here presented, into the forms and objects that may best fulfil his intentions, and meet the various exigencies of each particular occasion." *Preface.*

In the Historical Introduction, our author exhibits a rapid but very spirited sketch of the various causes, moral and physical which have operated in producing the extraordinary vicissitudes, which so eminently characterize the history of materia medica. The most prominent of these causes are classed and delineated under the heads—

"Superstition; Credulity; Scepticism; False Theory; Devotion to Authority and Established Routine; the assigning to Art that which was the Effect of unassisted Nature; Ambiguity of Nomenclature; the Progress of Botanical Science; the Application and Misapplication of Chemical Philosophy; the Influence of Climate; &c. The ignorant Preparation or fraudulent Adulteration of Medicines; the Obscurity which has attended the Operation of Compound Medicines." *Introduction*, P. ix.

In the discussion of each of these heads, our author displays considerable research, much critical acumen, and classical elocution—with occasional veins of keen satire. Under the head of Chemical Science, Dr. Paris takes an opportunity of defending, with much zeal and some warmth, the alma maters and collegiate institutions of his country.

"But from the very nature and object of a pharmacopœia, it cannot be supposed to proceed, *pari passu*, with the march of chemical science, indeed it would be dangerous that it should, for a chemical theory must receive the seal and stamp of experience before it can become current: a Pharmacopœia, however, is always an object of abuse, because it is a national work of authority, which is quite a sufficient reason why the ignorant and conceited should question its title to respect, and its claims to utility. '*Plures audivi*,' says Huxham, '*totas blaterantes Pharmacopœias, qui tamen ne intellexerint quidem quid vel ipse pulsus significabat.*'

"It is very evident, that the greater number of these attacks has not been levelled with any view to elicit truth, or to advance science, but to excite public attention, and to provoke unfair discussion for individual, and unworthy advantage; their obscure and presumptious authors vainly hope, that they may gain for their ephemeral writings some share of importance, and for themselves some degree of reputation, if they can only obtain notoriety, by provoking a discussion with the College, or with some of its responsible members, though such a combat should be sure to terminate in their defeat. Like the Scythian Abaris, who upon being wounded by Apollo, plucked the arrow from his side, and heedless of the pain and disgrace of his wound, exclaimed in triumph, that the weapon would in future enable him to deliver Oracles." *Introduction*, liv.

Dr. Paris avoids entering into discussion with this class; but warmly remonstrates with Professor Brande for an implied attack on the College, contained in a Preface to the Supplement of the Encyclopædia Britannica.

"It appears from Mr. Brande's laconic answer to Dr. Young, published in "*The Journal of Science and the Arts*," that his objections are those of Mr. Phillips, contained in his experimental examination of the Pharmacopœia, a work, which I confess, appears to me to furnish a testimony of the experimental tact, subtle ingenuity, and caustic style of criticism, which its author so eminently possesses, rather than a proof of any fatal or material inaccuracy in the Pharmacopœia; and I may urge this with greater force and propriety, when it is considered that at the time of its publication, I was not a Fellow of the College, and therefore had no voice upon the subject of its composition, and consequently must be *personally* disinterested in its reputation." *Introduction*, lvii.

Dr. Paris also protests against the prevailing fashion of examining and deciding upon the pretensions of every medicinal compound, by a mere chemical investigation of its composition, and of rejecting, as fallacious, every medical testimony which may appear contradictory to the laboratory. He instances GRIFFITH'S MIXTURE, as a medicinal combination which the ultra chemists of the day cried out against; but of which, subsequent experience has fully established the value, and that upon scientific principles.

"From a mistaken notion of this kind, the *Extractum colocynthidis compositum*, with a view of making it chemically compatible with *calomel*, has been deprived of the *soap* which formerly entered into its composition, in consequence of which, its solubility in the stomach is considerably modified, its activity is therefore impaired, and its mildness diminished." *Introduction*, lix.

We entirely agree with our enlightened author, that "the stomach has a chemical code of its own, by which the usual affinities of bodies are frequently modified, often suspended, and sometimes entirely subverted." The absurdity, indeed, of attempting to account for the phenomena of life (as Dr. Paris well observes) upon principles deduced from the analogies of inert matter, is felt in its full force by every rational physician, and the folly of the attempt is deprecated by the most intelligent physiologists of the present times.

The introductory essay, or "analysis of the objects to be attained by mixing and combining medicinal substances," contains so much original and important matter, that we must dwell some time on this interesting feature of the work. The following is the arrangement which our author lays down for this purpose.

"I. To promote the action of the basis, or principal medicine.

"A. By combining it with substances which are of the *same nature*, that is, which are *individually* capable of producing the same effects, but with less energy than when in combination with each other." *Key letter* a.

The above rule or principle is illustrated by many apposite and practical examples. Thus our author justly observes, that emetics are more efficient when ipecacuan and tartarized antimony, or sulphate of zinc are combined, than when exhibited separately, though in an equivalent dose. That the power of cathartics is increased by combination, while their irritating properties are lessened: thus the ex. col. comp. is much more active and manageable, and less irritating than any of its components separately taken.

" *Diuretics.* Under this class of medicinal agents it may be noticed, that *whenever a medicine is liable to produce effects different from those we desire, its combination with similar remedies is particularly eligible*, by which the action of the basis may be directed and fixed: thus the individuals which compose the class of Diuretics, are uncertain in their operation, and disposed, when exhibited singly, to produce diaphoretic, and other contrary effects: it is therefore in such cases, highly judicious to unite several of them in one formula, by which we increase their powers, and are more likely to insure their operation. Formulæ 29, 31, 36, 37, 38, 39, are constructed upon this principle." 2.

We cannot pursue our author's excellent observations on expectorants, diaphoretics, emmenagogues, stimulants, tonics, alteratives, &c. but refer to the volume itself, for further information.

Under this head, our author throws out several important hints respecting the *similarity of medicinal virtues* in the substances combined. Classification, alone, may lead us into many and dangerous errors. Experience must establish a perfect similarity in their operation, under every condition of the human body, and that by modes of action which are compatible with each other.

" Thus *squill*, *calomel*, and *digitalis*, are each powerful diuretics, but nevertheless they cannot be considered similar remedies, since *digitalis* will entirely fail in its effects in the very cases that *calomel* and *squill* succeed; and *squill* will prove inert, when *digitalis* is capable of producing the most powerful influence; this arises from their modes of operation being dissimilar, and consequently requiring for their success, different states of the living system.

" Dr. Blackall, in his ' Observations upon the Cure of Dropsies,' has offered some remarks so valuable in themselves, and so illustrative of this important subject, that I beg leave to quote the passage. ' Many physicians, he observes, are fond of combining *squill*, *calomel*, and *digitalis*, as a diuretic in dropsy; a practice often unsafe, and not very decidedly possessing the merit even of being consistent. Digitalis greatly depresses the action of the heart and arteries, and controls the circulation, and it seems most unreasonable to believe that its curative powers can be independent of such an effect; on the other hand, mercury, if it does not pass off quickly, is always exciting fever, and raising and hardening the pulse; speaking from experience, where the urine is coaguable, and *digitalis* agrees, both the others are, often at least, positively injurious. On the contrary, where the urine is foul, and not coaguable, and *squills* with *calomel* render service, I have on that very account made less trial of digitalis, and cannot therefore speak of it from much experience.' "

" B. By combining the basis with substances of a *different nature*, and which do not exert any chemical influence upon it, but are found by experience to be capable of rendering the stomach, or system, or any particular organ, more susceptible of its action." *Key letter* b.

Thus, our author observes, the system is rendered more susceptible to the influence of mercury, by combining it with antimony and opium; the stomach, in certain cases, is rendered more obedient to emetics by the previous exhibition of opium, &c. &c. "Upon the same principle, antimonial wine quickens the operation of saline cathartics; opium increases the sudorific powers of antimony; and the purgative operation of jalap is promoted by ipecacuan." Dr. Paris aptly illustrates his subject here by adverting to the effect of venesection in rendering the system more susceptible of certain impressions.

"Reiterated practice has taught me, that the system in a strong and healthy condition frequently offers a resistance to the operation of mercury, which is overcome the moment the stomach becomes deranged, the circulation languid, or the general tone of the system impaired. I have frequently seen this during my hospital practice: if a patient, who has been using mercurial friction, or taking the preparations of that metal without effect, be transferred into a close and unhealthy ward, his appetite soon fails, the tongue becomes furred, and the system instantly yields to the influence of the remedy. Nauseating doses of *antimony*, frequently repeated, or the accidental supervention of any disease of debility, will be attended with the same phenomena. My practice has also afforded me an opportunity of appreciating the debilitating effects of despondency in a case of this description; a patient had been taking mercurial medicines, and using frictions for a considerable period, without any apparent effect; under these circumstances he was abruptly told that he would fall a victim to his disease; the unhappy man experienced an unusual shock at this opinion, and in a few hours became violently salivated."

The effects of cathartic medicines are also increased by previous venesection; and purgatives themselves seem capable of exalting the efficacy, and accelerating the benefit of many alteratives, when previously administered.

"II. To correct the operation of the basis, by obviating any unpleasant effects it might be likely to occasion, and which would pervert its intended action, and defeat the objects of its exhibition.

*Key letter* c.

This Section of our author's analysis is illustrated by many excellent observations on corrigents; but our limits will not permit us to introduce more than the following extract.

"The virtues of the most important remedies are frequently lost, or much invalidated, for want of proper attention to the circumstances comprehended in this section. It may be almost admitted as an axiom, that whenever an alterative medicine acts with violence upon the primæ viæ, its energies are uselessly expended, and the objects of its exhibition defeated. So, again, diaphoretics, diuretics, and many other remedies, suffer a diminution in their effects, whenever they stimulate the stomach or bowels to excess. Guaiacum loses its anti arthritic, squill its diuretic, and antimony and ipecacuan their diaphoretic virtues under such circumstances. The action of these substances therefore requires correction, and a medicine must be selected capable of fulfilling that intention. Opium has very extensive powers as a corrigent. Formulæ 28, 34, 38, 89."

"III. To obtain the joint operation of two or more medicines, which have different powers, and which are required to obviate different symptoms, or to answer different indications." 13. *Key letter* d.

Among various illustratrations of the above Section our author brings forward the treatment of *cholica pictonum*, where it is found, that purgatives produce no effect unless the spasm be allayed by combining them with opium—of *dropsies*, when we have often to evacuate the water, and brace the solids at the same time.

"IV. To obtain a new and active remedy not afforded by any single substance.

"A. By combining medicines which excite different actions in the stomach and system, in consequence of which new or modified results are produced." *Key letter* e.

This is an obscure subject, in our present state of knowledge. That the effects above described, however, are really produced by such arrangements we have the testimony, Dr. Paris observes, of long experience, as is exemplified, for instance, in the *pulvis ipecacuanhæ compos.* which, in well regulated doses, is divested of the narcotic operation of the opium, and the nauseating effects of the ipecacuanha. The Plummer's pill offers a similar example, in respect to calomel and antimony.

"B. By combining substances which have the property of acting chemically on each other; the result of which is the formation of new Compounds, or the decomposition of the original constituents, and the developement of their more active Elements."

Of the numerous examples brought forward by Dr. Paris in illustration of the above, we can only cite one; namely, the formula of an elegant purgative draught created by chemical combination, in which the original properties of every element are entirely changed.

℞. Sodæ sub-carbonat. crystal. ʒiiß.

Crystallorum tartari - - ʒiij.

Aquæ puræ - - - - ℥viij.

Stent in lagena bene obturata per triduum, et deinde sit in promptu pro potu ordinario. *Young's Med. Liter. p.* 445.

"C. By combining substances, between which no other chemical change is induced than a diminution or increase in the solubilities of the principles, which are the repositories of their medicinal virtues." 19. *Key letter* h.

We shall not be able to do justice to this Subsection of our author, the subjects descanted on being numerous and important. The degree of solubility in a medicinal substance may, Dr. P. thinks, not only influence the activity of the remedy, but, like its dose, go far to determine its specific operation. Thus gamboge, by dissolving before it passes the pylorus, is liable to affect the stomach; aloes, on the contrary, on account of its slow dissolution, acts principally on the rectum, occasioning irritation there, if not counteracted by combination with soap and an alkaline salt which quicken its operation. The compound decoction of aloes affords a combination of this kind. Resinous purgatives are apt to gripe, from their slow solubility, which is prevented or removed by combining them with neutral salts. The addition of alkalies, or lime water to the infusions of gentian, &c. or decoctions of bark, by rendering their extractive and resinous principles more soluble, increase their elegance and exalt their virtue.

It appears, Dr. Paris thinks, to be established as a pharmaceutical axiom, that—

"*The intensity, and even specific action of a purgative medicine may be modified or changed, by changing the degree of solubility possessed by the principles in which its activity resides.*"

"In the exhibition of diurectics likely to become cathartic or diaphoretic, no liquid should be given, for at least an hour after their administration. The same caution applies with respect to the compound powder of ipecacuan, which has a strong tendency to excite vomiting. When the remedy has passed out of the stomach, then the ingestion of fluids may, and ought to be encouraged." 23.

Many highly interesting observations follow on the counteraction of poisons, for which we must refer to the Section in question, which indeed contains a great deal of original and ingenious matter.

"V. To afford a convenient, agreeable, and efficacious form. *Key letters* i. k.

"The perfection of a medicinal prescription may be defined by three words; it should be Precise (in its directions), Concise (in its construction), Decisive (in its operation). It should carry upon its very face an air of energy and decision, and speak intelligibly the indications which it is to fulfil. It may be laid down as a position, which is not in much danger of being controverted, that where the intention of a medicinal compound is obscure, its operation will be imbecile."

Dr. Paris divides medicinal formulæ into four constituent parts, viz. 1st. The basis or principal medicine. 2d. The adjuvant, to promote its operation. 3tio. The corrigens; and 4to. The constituent, or that which imparts an agreeable form.

We must here pass over a large space of the work, full of important matter, to give some account of Dr. Paris's disposition of the key letters, placed opposite to the elements of the formulæ, which furnish the practitioner with a farther elucidation of the foregoing principles.

These key letters will immediately explain themselves, if the reader will turn back and compare them with the letters placed in each of the five sections, which we have just sketched out. We shall, however, exemplify their use by reference to a single formula in particular.

Formula 16. Cathartics.

| | | Key Letters. |
|---|---|---|
| ℞. | Pulv. aloes comp. ʒj. | |
| | Pulv. antimon. gr. v. | b. |
| | Sapon. duri gr. x. | i. h. |

Decoct. aloes. comp. q. s. fiat massa in pilulas xx dividenda.

If the reader refers to the key letter b. he will see that experience has shewn that the pulvis antimonialis, in the above prescription, renders the stomach or system more susceptible of the action of the basis, aloes. The letters i and h will shew that the soap promotes the solubility of the aloes, and renders the form agreeable.

Dr. Paris gives us, in this department of his work, one hundred and twenty-five select prescriptions, many of them taken from the best practical writers, exemplifying the various principles inculcated or set forth in his book. We dare not stop to copy any of them, as we perceive that our bounds are likely to be greatly exceeded.

Into the PHARMACOLOGIA, or great body of the work, containing a concise, yet comprehensive commentary, medical, chemical, and philosophical, on each article, simple and compound, of the Pharmacopœia, we cannot be expected to enter, in an *analytical* manner, since this part of the work is, in itself, a concentrated analysis. Yet, as an inducement for all those who have not the publication to quickly possess themselves of it, we shall string together a rosary, not of flowery extracts from the work, but merely of *alphabetical* specimens, noted down almost at random, and which may convey a faint, yet satisfactory idea of the whole composition.

"Ex pede Herculem."

1. *Acidum muriaticum,* says our author, after a copious evacuation from the bowels, is, in my experience, the best remedy for preventing the generation of worms; for which purpose, the infusion of quassia, stronger than that of the Pharmacopœia, is the best vehicle. Dose, M. x. to L. frequently repeated. 84.

2. *Aloes.* Its solubility is materially increased by alkalies and soaps; but *then* it is liable to dissolve in the stomach, and occasion sickness, &c. Uncompounded with those articles, it does not dissolve till after it passes the stomach, and thus imitates the action of the bile on the intestines. Where it is desirable to exhibit aloes, and yet the patient is subject to hæmorrhoids, the foregoing medicines may be advantageously combined.

"Anderson's pills consist of aloes, with a proportion of jalap and oil of aniseed—Hooper's Pills, pil. aloes cum myrrha, sulphate of iron, and canella bark — Dixon's Anti-bilious Pills, aloes, scammony, rhubarb, and tartarized antimony—Speediman's Pills, aloes, myrrh, rhubarb, extract of chamomile, and essential oil of chamomile. Dinner Pills—Lady Webster's or Lady Crespigny's Pills, ℞. Aloes opt. ℥vj; masticbes et rosarum rubrarum āā ʒij, syrupi de absynthio q. s. ut fiat massa. The mass is divided into pills of three grains each. The operation of this pill is to produce a copious and bulky evacuation; and in this respect, experience has fully established its value." 98.

3. *Amygdalæ Amaræ.* "M. Majendie has lately published some Essays on the Use of Prussic Acid in Pulmonary Consumption; there is, however, nothing new in its application in such cases, for Linnæus informs us, that distilled laurel water was frequently used in Holland in pulmonary consumption." *Amœnitat. Acad. vol. iv.*

4. *Antimonium Tartarizatum.* One fluid ounce of the decoction of yellow bark is capable of completely decomposing ℈j. of tartarized antimony, and rendering it inert. Berthollet has accordingly recommended the immediate exhibition of this decoction, when an overdose of the salt has been taken.

"Rhubarb is equally incompatible. The extract of this substance, therefore, ought never to be employed in forming pills of emetic tartar." 116.

5. *Arsenic.* After stating the test for the presence of arsenic, published in the Philosophical Magazine, by Mr. Hume of London, our author gives what he considers an improvement in the application of the silver test, which we shall here introduce.

"My method consists in conducting the trial on writing paper, instead of in glasses, thus—drop the suspected fluid on a piece of white paper, making with it a broad line; along this line a stick of lunar caustic is to be slowly drawn several times successively, when a streak is produced of a colour resembling that known by the name of *Indian Yellow;* and this is equally produced by the presence of arsenic and that of an alkaline phosphate, but the one from arsenic is rough, curdy, and flocculent, as if effected by a crayon, that from a phosphate homogeneous and uniform, resembling a water colour laid smoothly on with a brush; a more important, and distinctive peculiarity soon succeeds, in less than two minutes the phosphoric yellow fades into a *sad green*, and becomes gradually darker, and ultimately, quite black; the arsenical yellow, on the other hand, remains permanent, or nearly so, for some time, when it becomes brown. In performing the experiment, the sun-shine should be avoided, or the transitions of the colour will take place too rapidly. It would be prudent also for the inexperienced operator to perform a similar experiment on fluids known to contain arsenic, and a phosphoric salt, as a standard of comparison. In this way, the nitrate of silver, without the intervention of any other test, is fully capable of removing every ambiguity, and of furnishing a distinguishing mark of difference between the chemical action of arsenic and the phosphates." 139.

6. *Balsam of Copaiba.* This article is too often adulterated. M. Bucholz asserts, that if it does not dissolve in a mixture of four parts of pure alcohol, and one of rectified ether, we may infer its adulteration. 187.

7. *Barclay's Anti-Bilious Pills.* Take of extract of colocynth ʒij, extract of jalap ʒj, almond soap ʒiß, guaiacum ʒiij, tartarized antimony grs. viij, essential oil of juniper, carraway, and rosemary, of each four drops; syrup of buckthorn q. s. to make sixty-four pills.

8. *Elaterium.* Our readers are aware that Dr. Clutterbuck has lately detailed in the London Medical Repository, a Series of interesting and instructive Experiments on Elaterium, proving satisfactorily "that the active principle of this plant (cucumber) is neither lodged in the roots, leaves, flowers, nor stalks, *in any considerable quantity;* nor is it to be found in the body of the fruit itself, or in the seeds, but in the *juice around the seeds*." The substance spontaneously subsiding from this liquor, obtained without pressure, is *genuine* elaterium; the quantity of which, contained in the fruit, is so small, that Dr. Clutterbuck obtained only six grains from forty cucumbers. Dr. C. having communicated these details to the president of the College of Physicians, Dr. Paris, as Professor of Materia Medica to the College, was requested by the President to Report upon them. The results of Dr.

Paris's experiments (amply detailed in the work before us) shew that not more than *one grain in ten* of elaterium, as it occurs in commerce, possesses any active properties! This decimal part is a vegetable proximate principle, not hitherto noticed, to which Dr. Paris has given the name of *Elatin*, authorized, he thinks, by the results of the experiments detailed in pages 223, 4, of the Pharmacologia.

*Chemical Composition of Elaterium.*

| | |
|---|---|
| Water | .4 |
| Extractive | 2.6 |
| Fæcula | 2.8 |
| Gluten | .5 |
| Woody matter | 2.5 |
| Elatin, bitter principle | 1.2 |
| | 10 grs. |

The dose of good elaterium, as it occurs in commerce, is about two grains; "or it is better to give it only to the extent of half a grain at a time, and to repeat that dose every hour until it begins to operate. It is, probably, when thus managed, the best hydragogue cathartic which we possess." 225.

9. *Hydrargyrum cum Creta.* We think this preparation of mercury too much neglected, and coincide fully with Dr. Paris in the following sentiments.

"It is a mild and excellent mercurial, and has been known to cure syphilitic affections, when the constitution had proved rebellious to every other form of preparation. Dr. George Fordyce committed a great error, when he denied to this compound any mercurial efficacy." 244.

10. *Hydrargyri Oxy-Murias* generally forms the basis of those dangerous nostrums which are advertised for the cure of syphilis *without mercury.* Gowland's lotion is a solution of *sublimate* in an emulsion of bitter almonds, gr. iſs to f.℥j.—Spilsbury's Antiscorbutic Drops, Dr. Paris states to be ʒij of sublimate, prepared sulphuret of antimony ʒj, gentian root and orange peel aa ʒij, shavings of red Saunders ʒj, made into a tincture, with a pint of proof spirit.—Soloman's Anti-Impetigines, a solution of sublimate.—Velno's Vegetable Syrup has not been accurately ascertained. It is supposed (says Dr. P.) to be sublimate rubbed up with honey and mucilage. Our author has reason to believe that it contains *antimony,* and the syrup of marsh mallows. The volatile alkali was proposed by Dr. Peyrile, as a substitute for mercury; and Swediaur says, that it is an ingredient in this nostrum.

"Caution. The salt, (sublimate) as it is partially decomposed by light, should be kept in opake bottles.—Adulterations. It ought to be volatilized by heat; it is frequently met with in commerce, contaminated with muriate of iron, sometimes with arsenic; the presence of calomel is at once discovered from its insolubility.—Tests of its presence. If any powder be suspected to contain this salt, expose it to heat, in a coated tube, as directed in the treatment of arsenic, but without any carbonaceous admixture, when corrosive sublimate, if present, will rise and line the interior surface with a shining white crust. This crust is then to be dissolved in distilled water, and assayed by the following tests: 1st. *Lime water* will produce, if the suspected solution contain this salt, a precipitate of an orange yellow colour. 2d. A single drop of a dilute solution of *sub-carbonate of potass* will at first produce a white precipitate, but on a still farther addition of the test, an orange-coloured sediment will be formed. 3d. *Sulphuretted water* will throw down a dark coloured precipitate, which, when dried and strongly heated, may be volatilized without any alliaceous odour." 249.

11. *Liq. Antim. Tart.* During the time that Dr. Paris was Censor of the College, he and his colleagues took pains to ascertain the state in which this preparation obtained throughout the wholesale and retail shops of the metropolis.

"We were satisfied, during our official visitations, that where *sound* Sherry wine had been employed as a solvent, an efficient and permanent solution was obtained, and that no precipitation of the antimony took place, the sediment which occurred being merely *tartrate of lime*, an incidental impurity, derived from the *cream of tartar*, of the tartarized antimony; but in a majority of instances, an inferior wine was substituted, in which case the antimonial oxyd was found in a copious precipitate, in combination with vegetable extractive matter; and I have since seen this decomposition so complete, that the super-natant liquor would not yield any trace of the antimonial salt, when assayed with sulphuret of potass. Under such circumstances, it might be more judicious to remove *antimonial wine* from the list of *officinal* preparations." 274.

12. *Liquor Potassæ.* Under this head, Dr. Paris is naturally led to the important subject of urinary calculi, from which modern chemistry has now almost drawn aside the veil of obscurity with which it was so long covered. The varieties of calculi produced by the combination, or intermixture of lithic or uric acid, phosphate of lime, ammoniaco-magnesian phosphate, oxalate of lime, cystic oxyd, and animal cementing ingredients, are well represented in the following tabular arrangement, which we shall here introduce from Dr. Paris's work.

### *A Tabular View of the different Species of Urinary Calculi.*

| SPECIES OF CALCULI. | EXTERNAL CHARACTERS. | CHEMICAL COMPOSITION. | REMARKS. |
|---|---|---|---|
| 1. LITHIC or URIC. | *Form*, a flattened oval; *specific gravity*, generally exceeds 1·500; *colour*, brownish or fawn-like; *surface* smooth; texture laminated. | It consists, principally, of *Lithic acid*; when treated with nitric acid, a beautiful pink substance results. This calculus is slightly soluble in water, abundantly, in the pure alkalies. | It is the prevailing species; but the surface sometimes occurs finely tuberculated. It frequently constitutes the *Nuclei* of the other species. |
| 2. MULBERRY. | *Colour*, dark brown; *texture*, harder than that of the other species; *sp. grav.* from 1·428 to 1·976; *surface*, studded with tubercles. | It is *Oxalate of Lime*, is decomposed in the flame of a spirit lamp, swelling out into a white efflorescence, which is *Quick Lime*. | This species includes some varieties, which are remarkably smooth, and pale coloured, resembling a *hemp seed*. |
| 3. BONE EARTH. | *Colour*, pale brown or grey; *surface* smooth and polished; *structure*, regularly laminated; the laminæ easily separating into concrete crusts. | Principally *Phosphate of Lime*. It is soluble in muriatic acid. | |
| 4. TRIPLE. | *Colour*, generally brilliant white; *surface* uneven, studded with shining crystals; less compact than the preceding species; between its laminæ small cells occur, filled with sparkling particles. | It is an *Ammoniaco-magnesian Phosphate*, generally mixed with phosphate of lime; pure alkalies decompose it, extricating its ammonia. | This species attains a larger size than any of the others. |
| 5. FUSIBLE. | *Colour*, greyish white. | A compound of the two foregoing species. | It is very fusible. melting into a vitreous globule. |
| 6. CYSTIC. | Very like the Triple Calculus, but it is unstratified and more compact, and homogeneous. | It consists of *Cystic Oxide*; under the blow-pipe, it yields a peculiarly fœtid odour. It is soluble in acids, and in alkalies, even if they are fully saturated with carbonic acid. | It is a rare species. |
| 7. ALTERNATING. | Its section exhibits different concentric laminæ. | Composed of several species, alternating with each other. | |
| 8. COMPOUND. | No characteristic form. | The ingredients are separable only by chemical analysis. | |

Dr. Paris here introduces several important practical observations on the treatment of calculous affections, for which we refer to page 281, *et Sequent.*

13. *Dalby's Carminative.* ℞. Carb. magnes. ℈ij; ol. menth. pip. m. i; ol. nucis mosch. m. ii; ol. anisi, m. iij; tinct. castorei, m. xxx; tinct. assafœtidæ, m. xv; spir. pulegii, m. xv; tinct. card. comp. m. xxx; aq. menth. pip. ℥ij. M.

Dr. Paris observes that this medicine is constructed upon philosophical principles, and therefore a similar composition may occasionally be prescribed by the regular medical practitioner with advantage.

14. *Mistura Ferri Compos.* "This is nearly the same as the celebrated anti-hectic mixture of Dr. Griffith; to the result of the decompositions which take place from the mixture of its ingredients, it is wholly indebted for its medicinal energies, thus, a *proto-carbonate of iron* is formed, i. e. the iron, combined with carbonic acid, is at its *minimum* of oxidation, which renders it more active than the common carbonate, and probably less stimulant than the sulphate; this product, by means of the saponaceous compound formed by the union of the myrrh with the excess of alkali, is *partly* diffused and suspended in the mixture, and *partly* dissolved, whilst, at the same time, a *sulphate of potass* is formed, which serves to correct the astringent influence which iron is apt to exert upon the bowels. The iron in this preparation is disposed to combine with an additional proportion of oxygen, hence its ingredients should be quickly mixed together, and it ought to be considered as an extemporaneous preparation; it should be preserved in a closely-stopt vessel. Its change of colour will generally indicate its loss of efficacy.

"*The dose* of the above mixture is f ʒj to f ʒij twice or thrice a day." 295.

15. *Plumbi Superacetas.* Dr. Paris feels no hesitation in pronouncing this salt of lead to be one of the most valuable resources of physic. From the results of numerous cases he can state with confidence, that it is more efficient in stopping pulmonary and uterine hæmorrhage than any other known remedy, and that its application is equally safe and manageable. We ourselves have found it extremely efficacious in hæmoptysis, especially when combined with opium. It should not be given, of course, in infusion of roses, or with sulphate of magnesia, as is sometimes the case.

16. *Potassæ Acetas.* Under this head, Dr. Paris introduces some interesting observations relative to the action of the digestive organs on certain medicines of the diuretic class.

"I beg therefore (says he) to observe, that the results of experiments, carefully conducted, and of observations faithfully recorded, during the exercise of my profession, have fully satisfied me that the digestive organs possess the power of readily decomposing all saline compounds, into which *vegetable* acids enter as ingredients, and of eliminating their alkaline base, which, being in the course of circulation carried to the kidnies, excites them into action, and promotes the excretion of urine; and that it is in this way, the *acetate, citrate, super-tartrate,* and other combinations of potass, or soda, prove diuretic: on the other hand, it is equally evident, that salts containing the *mineral* acids are not under the control of the decomposing powers of the *chylo-poietic* organs, and consequently do not undergo any changes *in transitu*; although some of these salts, especially the more soluble ones, are absorbed, and stimulate the urinary organs by their contact; this happens with *nitrate of potass,* which may be chemically detected in the urine of those persons who have taken it." 233, 334.

Our author also states, that the diuretic operation of any saline body that acts by absorption, is at once suspended if a catharsis be induced, either by the largeness of the dose, its increased solubility, or its combination with any other remedy:

" For it is a law that the processes of assimilation and absorption are arrested, or very imperfectly performed during any alvine excitement."

This, he thinks, is well illustrated by the different effects of the saline compounds of the alkalies with tartaric acid. Thus cream of tartar, in well regulated doses, acts, as we all know, upon the kidnies; the tartaric acid being, in this case, abstracted and assimilated by the digestive process, and the alkaline base at the same time eliminated, and subsequently absorbed.

" But if we increase the solubility of the compound, by reducing it to the state of a neutral tartrate (soluble tartar) or by combining it with boric acid, or some body that has a similar effect; or what is equivalent to it, if we increase the dose of the cream of tartar, that catharsis follows its administration, then diuresis will not ensue, since no decomposition can, under such circumstances, take place." 335.

We confess that, to our minds, this physiology appears rather too chemical. We can very easily conceive, that when the mucous membrane of the intestines is briskly acted upon, the secretion of urine will be diminished, without any recourse to chemical decomposition. In fact, we find this to be the case when a *spontaneous* diarrhœa takes place, and, of course, where chemical changes cannot be operating. We agree with our author, however, " that by associating saline diuretics with substances capable of rendering the powers of digestion more acute, we should be more likely to ensure their successful operation." This indeed is proved by daily experience; "for alkalies and their compound salts are never more active than when exhibited in combination with vegetable bitters."

"For the same reason, although purgatives, when *simultaneously* exhibited with this class of diuretics, will invalidate their powers, they may, nevertheless, when previously exhibited, be the means of assisting their operation; for it is probable that the absorbents are more ready to take up medicinal bodies, after a full evacuation of the bowels." 335.

17. *Pulvis Antimonialis.* The following is the composition of James's Analeptic Pills." ℞. Pulv. jacobi, gum ammon. pil. aloes, c. myrrha, partes equales; tinct. castorei, q. s. ad faciendum massam.

18. *Sodæ Carbonas.*

"*Sodaic Powders.*—Contained in two distinct papers, one of which is blue, the other white; that in the former consists of ʒss of the *carbonate of soda*, that in the latter, of grs. xxv. of *tartaric acid*. These powders require half a pint of water. It is very evident that a solution of these powders is by no means similar to '*soda water*,' which it is intended to emulate; for in this latter preparation, the soda is in combination only with carbonic acid; whereas, the solution of the '*sodaic powders*' is that of a neutral salt, with a portion of fixed air diffused through it.

"*Patent Seidlitz Powders.*—These consist of two different powders; the one, contained in a white paper, consists of ʒij of *tartarized soda*, and ℈ij of *carbonate of soda;* that, in the blue paper, of grs. xxxv of tartaric acid. The contents of the white paper are to be dissolved in half a pint of spring water, to which those of the blue paper are to be added; the draught is to be taken in a state of effervescence. The acid being in excess renders it more grateful, and no less efficacious, as a purgative." 369.

19. *Cheltenham Salts.* Dr. Paris informs us that a factitious compound, as a popular purgative, under the above name, has long been vended.

"It is formed by triturating together the following salts: *Sulphate of soda*, grs. 120; *sulphate of magnesia*, grs. 66; *muriate of soda*, grs. 10; *sulphate of iron*, grs. ½. As a purgative it is very efficacious, and superior, probably, to that which is actually obtained by the evaporation of the Cheltenham water itself, for, notwithstanding the high pretensions with which it has been publicly announced, it will be found to be little else than common *Glauber's salt*." 373, 374.

We have now offered sufficient specimens of the *matter* of Dr. Paris's work; it yet remains to give a single short specimen of the *manner* in which his pharmacologic articles are constructed. We extract the following, on account of its conciseness, as we have considerably overrun the limits which we originally chalked out for this Review.

" TARAXACI RADIX. L. E. (Leontodon Taraxacum) *Dandelion.*

" QUALITIES.—*Odour*, none; *taste*, bitter, and somewhat sweet, and acidulous.—CHEMICAL COMPOSITION. The active principles appear to consist of extractive, gluten, a bitter principle *(not resinous)*, and tartaric acid.—SOLUBILITY. Water extracts its virtues much better than spirit.—INCOMPATIBLE SUBSTANCES. *Infusion of galls*, *nitrate of silver*, *oxy-muriate of mercury*, *superacetate of lead*, and *sulphate of iron*, occasion precipitates in its solutions.—MED. USES. It has long enjoyed the reputation of proving beneficial in obstructions of the liver, and in visceral diseases; Bergius extols its use in these complaints, and recommends the recent root to be boiled in whey or broth. Dr. Pemberton has more recently added his testimony to its value; he observes, that he has seen great advantage result from using the extract in chronic inflammation, and incipient schirrus of the liver, and in chronic derangement of the stomach.—FORMS OF EXHIBITION. In that of extract, or in decoction, made by boiling ʒj of the sliced root in oj of water, down to oss, adding to the strained liquid ʒj of cream of tartar; the recent full grown root only should be used.—DOSE f℥iij. twice or thrice a day.* OFFICINAL PREP. *Extract. taraxaci*, The roots are roasted, and used at Gottingen, by the poorer people, for coffee, from which, a decoction of them properly prepared can hardly be distinguished. The leaves of this plant are blanched, and very commonly used on the continent as a sallad." 390, 391.

We shall conclude this long analysis with a single remark: We have placed the PHARMACOLOGIA on the select shelf of daily reference, in the *practical* wing of our library, surrounded by a few honourable specimens of human industry and talent, and over which is inscribed the following motto, reversed from the Roman satirist.

horum
Nunquam ego optarim pauperimus esse librorum.†

---

* We are much in the habit of prescribing the decoct. taraxaci; but instead of one dr. we order one oz. for the above quantity of water, exhibiting a wine glassful of the decoction twice or thrice a day. Rev.

☞ Since writing the above, we find that for *drachms* we ought to read *ounces* in the above extract from Dr. Paris's work.

† horum
Semper ego optarim pauperimus esse bonorum. HOR.

